# Structural Characterization of Ordered Mesoporous Silica Prepared by a Sol–Gel Process Using Urea-Based Cationic Gemini Surfactants

**DOI:** 10.3390/gels11100804

**Published:** 2025-10-07

**Authors:** Sarvarjon Kurbonov, Zsolt Czigány, Zoltán Kovács, László Péter, Martin Pisárčik, Miloš Lukáč, Manfred Kriechbaum, Vasyl Ryukhtin, Ana-Maria Lacrămă, László Almásy

**Affiliations:** 1 Institute for Energy Security and Environmental Safety, HUN-REN Centre for Energy Research, Konkoly-Thege Miklós út 29-33, 1121 Budapest, Hungary; sarvarjon@student.elte.hu; 2 Doctoral School of Physics, Faculty of Natural Sciences, Eötvös Loránd University (ELTE), Pázmány Péter sétány 1/A, 1117 Budapest, Hungary; 3 Institute of Technical Physics and Materials Science, HUN-REN Centre for Energy Research, Konkoly-Thege Miklós út 29-33, 1121 Budapest, Hungary; 4 HUN-REN Wigner Research Centre for Physics, Konkoly-Thege Miklós út 29-33, 1121 Budapest, Hungary; 5 Department of Chemical Theory of Drugs, Faculty of Pharmacy, Comenius University, SK-83232 Bratislava, Slovakia; 6 Institute of Inorganic Chemistry, Graz University of Technology, 8010 Graz, Austria; 7 Nuclear Physics Institute, Czech Academy of Sciences, 250 68 Husinec—Řež, Czech Republic; 8 “Coriolan Drăgulescu” Institute of Chemistry, Bv. Mihai Viteazul, No. 24, 300223 Timisoara, Romania

**Keywords:** MCM-41, TEM, SEM, SAXS, micelle, nanoparticle

## Abstract

Mesoporous silica nanoparticles have been synthesized through sol–gel synthesis in basic conditions. Gemini surfactants having urea in the headgroups were used as pore-forming agents. The effect of the spacer length of the surfactant on the particle morphology was studied on the sub-micrometer and nanometer scales using nitrogen porosimetry, small-angle X-ray scattering (SAXS), ultra-small-angle neutron scattering, and scanning and transmission electron microscopy (SEM, TEM). Depending on the spacer, spherical and/or cylindrical nanoparticles formed in different proportions, as revealed by statistical analysis of SEM micrographs. All prepared materials showed the hexagonal pore structure characteristic of the MCM-41 molecular sieves, with the exception of the sample prepared using the gemini surfactant with the shortest spacer length. The influence of the spacer length on the lattice parameter of the pore network, as well as the average size of the ordered domains, has been assessed by SAXS and TEM. Detailed analysis of the TEM images revealed a spread of the lattice parameter in a range of 10–20%. The broadening of the diffraction peaks was shown to be due to the combination of the effects of the finite domain size and the variance of the lattice parameter across the crystalline domains. The structural differences between the silica gels synthesized with the different surfactants were related to the variation of the micelle morphologies, reported in previous light scattering and small-angle scattering experiments. No connection could be revealed between the micelle shape and size and the pore sizes, showing that surfactants with a broad range of spacer lengths can equally well be used for the preparation of MCM-41 materials.

## 1. Introduction

Nanomaterials, characterized by at least one of their dimensions smaller than 100 nm, have attracted substantial research interest due to their adjustable macroscopic properties. These properties are strongly influenced by factors such as size, shape, conditions of synthesis, and functionalization. Porous nanomaterials with large specific surface areas and well-defined nanostructures are especially useful in various fields, including adsorption, separation, heterogeneous catalysis, gas storage, and sensor technologies [1,2]. The synthesis and functionalization of these materials have become focal points in research because of their potential applications in advanced technological and scientific fields [3,4,5,6,7].

Among nanostructured porous materials, ordered mesoporous silica (OMS) stands out due to its uniform pore distribution, high thermal stability, and tunable surface characteristics. MCM-41, a member of the Mobil Composition of Matter (MCM) family, is especially notable for its ordered hexagonal pore structure, high specific surface area, and versatile synthesis approaches [4,5,8]. The tunable nature of MCM-41’s pore size, morphology, and surface chemistry facilitates its broad utilization in catalysis, drug delivery systems, and adsorption-based technologies.

The characteristic pore structure in OMS is formed in the wet synthesis during the condensation of the silica surrounding the micelles. Cationic surfactants, such as cetyltrimethylammonium bromide (CTAB), are the most commonly employed pore-forming agents. Recently, surfactants with more complex structures, in particular, gemini surfactants, have gained notable attention as potent structure-directing agents for fabricating mesoporous materials. Gemini surfactants comprise two polar head groups and two alkyl tails linked by a spacer near or directly at the head groups. This distinctive molecular arrangement offers enhanced surface activity, lower critical micelle concentrations (CMC), improved wetting and solubilization characteristics, and a higher likelihood of forming diverse aggregate structures compared to conventional monovalent surfactants [9,10]. Depending on the nature of the spacer chain and alkyl tail length, a wider variety of micellar aggregates, including elongated or disk-like shapes, can form in their aqueous solutions, affecting the morphology, pore structure, and wall thickness of the condensed silica network.

Numerous studies have confirmed the efficacy of gemini surfactants in synthesizing mesoporous silica materials. For instance, Voort et al. [11] utilized surfactants with the structure [C_n_H_2n+1_N^+^(CH_3_)_2_–(CH_3_)_s_–N^+^(CH_3_)_2_C_m_H_2m+1_]∙2Br^−^ as templating agents to produce OMS under alkaline conditions, showing that the surfactant of 16-8-16 type specifically directs the formation of MCM-41 with quality comparable to materials made using CTAB. Yang et al. [12] synthesized MCM-41 mesoporous molecular sieves using various gemini surfactants as templates. Among them, the material prepared with a self-designed asymmetric gemini ionic liquid surfactant exhibited the highest surface area and the most effective dye adsorption capability. It achieved an adsorption capacity of 464 mg/g for crystal violet under optimal conditions. These findings highlight the promising application of such mesoporous materials in environmental dye removal. Wang et al. [13] explored how varying lengths of poly(methylene) spacer chains of gemini surfactants influence the pore sizes, revealing that longer spacers significantly reduce the pore diameters in silica structures. Nitrogen adsorption/desorption analysis revealed the appearance of two mesoporosity types in the samples: dominant framework-confined pores and minor textural pores.

Previous investigations have also examined the effects of changing alkyl chain lengths and spacer groups in symmetric gemini surfactants, aiming at tailored synthesis of mesoporous silica [12,13,14,15,16]. Diester-based biodegradable gemini surfactant micelles were used in alkaline conditions to obtain MCM-41 particles with high surface areas and hollow sphere morphology. Structural characterization employing electron microscopy and low-angle diffraction revealed a hierarchical structure consisting of fragmented crystalline domains, which contributed to the high adsorption capacity of 113 mg/g for Pb(II) ions, presenting valuable environmental advantages [15]. Furthermore, Kaczerewska et al. [16] synthesized silica nanocapsules using innovative gemini surfactants, significantly reducing the toxicity of these nanocapsules toward marine organisms. Consequently, these developments allow the creation of environmentally friendly engineered nanomaterials (ENMs), consistent with the principles of Green Chemistry and a safe-by-design approach [17].

The current research thus focuses on synthesizing MCM-41 using symmetric urea-based gemini surfactants as pore templates. Incorporating urea functionalities into gemini surfactants introduces hydrogen bonding interactions between the neighboring molecules, which can distinctly influence micelle formation and subsequent pore structures compared to other spacer chemistries [18]. By systematically modifying spacer length, this study aims to evaluate its effect on the morphology and textural characteristics of the resulting OMS. The synthesized materials were thoroughly analyzed on length scales from nanometers up to micrometers using nitrogen physisorption, small-angle X-ray and neutron scattering, and electron microscopy.

## 2. Results and Discussion

### 2.1. Samples

**Figure 1:**
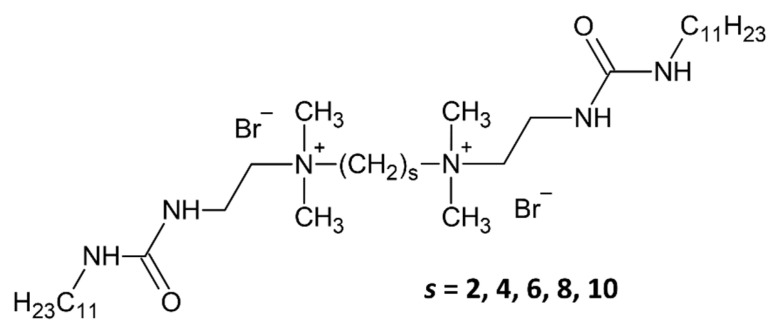
Molecular structures of the five urea-based gemini surfactants. The number of methylene groups in the spacer chain is denoted by “***s***”.

A series of urea-based gemini surfactants was employed as structure-directing agents to prepare ordered mesoporous silica materials with an ordered pore structure. The surfactants varied in their spacer chain length (the number of methylene units in the spacer denoted by “***s***”, see Figure 1), which influences their micellar assembly and pore templating behavior [11,18].

The mesoporous silica materials were synthesized in a sol–gel process using the Stöber method modified for the preparation of OMS [19,20]. The samples obtained after drying at 100 °C and those calcined at 540 °C were denoted as Us-100 and Us-540, respectively.

### 2.2. Infrared Spectroscopy

**Figure 2:**
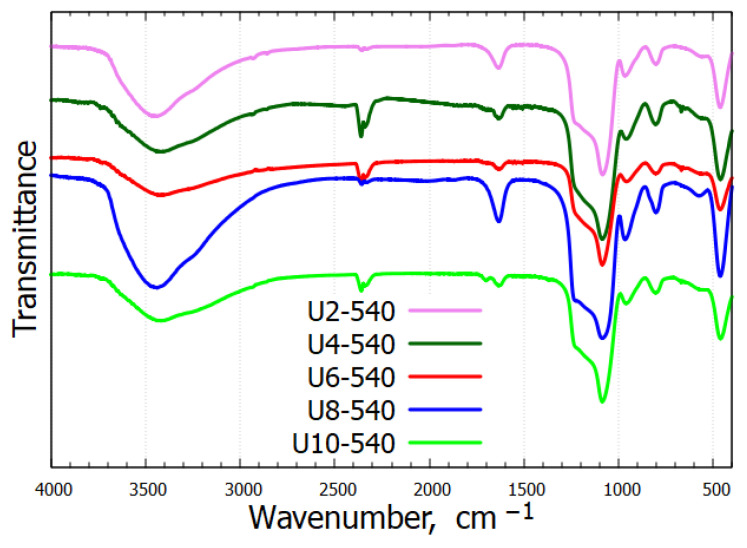
Infrared spectra of calcined mesoporous silica samples.

Figure 2 presents the infrared spectra of the calcined samples. A broad absorption band near 3455 cm^−1^ arises from the O–H stretching vibrations of hydrogen-bonded water molecules and surface Si–OH groups. Additionally, the band around 1636 cm^−1^ is the bending vibration of water molecules. The characteristic C–H stretching vibrations from the alkyl chains, typically appearing in the region of 2850–3000 cm^−1^, were absent in the calcined samples, confirming the complete removal of the surfactant. All samples exhibited characteristic bands of the silica framework near 1088 cm^−1^, 806 cm^−1^, and 463 cm^−1^. A distinct band around 960 cm^−1^, corresponding to the stretching vibrations of surface Si–OH groups, further confirmed the presence of silanol groups. The full list of band assignments is provided in Table 1. All observed IR bands support the successful preparation and calcination of silica.

### 2.3. Microscale Structure

#### 2.3.1. Scanning Electron Microscopy

**Figure 3:**
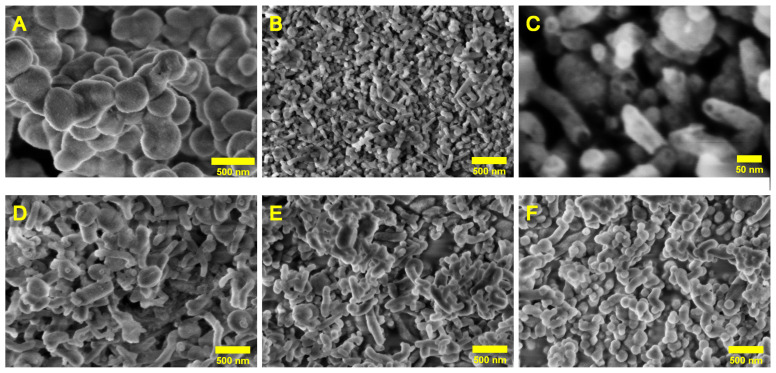
SEM images of the studied silica nanoparticles: (**A**) U2-100, (**B**,**C**) U4-100, (**D**) U6-100, (**E**) U8-100, (**F**) U10-100.

The morphology and size distribution of the synthesized samples (U2-100, U4-100, U6-100, U8-100, and U10-100) were analyzed by SEM, and characteristic images are shown in Figure 3. Spherical and cylindrical or tubular particles were found in all samples, with sizes below or around 1 micrometer. The ratios of the spherical and cylindrical particles were markedly different in the five samples. At larger magnifications, some of the cylindrical particles showed hollow interiors (see e.g., the U4-100 sample in Figure 3C), suggesting a tubular structure. For U2-100, all observed particles exhibited a spherical morphology, with diameters broadly distributed between approximately 100 and 800 nm. The U4-100 and U6-100 samples displayed mixed morphologies, each containing roughly equal proportions of spherical and cylindrical particles. These two samples exhibited a wide range of particle sizes, indicating higher polydispersity compared to U2-100. The U8-100 and U10-100 samples showed a mixture of morphologies but with different dominant shapes. In U8-100, cylindrical structures were more prevalent, whereas in U10-100, spherical particles dominated. The distribution of diameters of spherical particles and the thicknesses of the cylindrical particles were calculated using the ImageJ software (version 1.54p), and the approximate percentages of spherical and cylindrical particles were estimated by counting the number of each particle type from representative images of each sample. For all samples containing cylindrical particles (U4-100, U6-100, U8-100, and U10-100), the lengths of the cylinders could not be precisely measured due to overlapping, tilting, and curvature observed in the SEM images.

**Figure 4:**
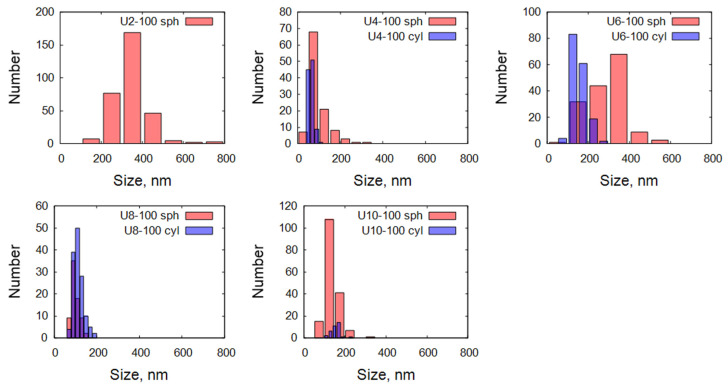
Distributions of diameters of spherical and cylindrical particles. “sph” stands for spherical, and “cyl” stands for the diameter of cylindrical particles.

A more detailed comparison of the size distributions in the five studied samples is shown in Figure 4. Overall, the SEM analysis revealed a broad particle size distribution across all samples, and the presence of spherical and cylindrical particles at various proportions. A brief summary of the observed morphologies is given in Table 2. Samples with mixed morphologies demonstrated particularly strong size broadening, attributed to the combination of different particle shapes and sizes.

#### 2.3.2. Ultra-Small-Angle Neutron Scattering

**Figure 5:**
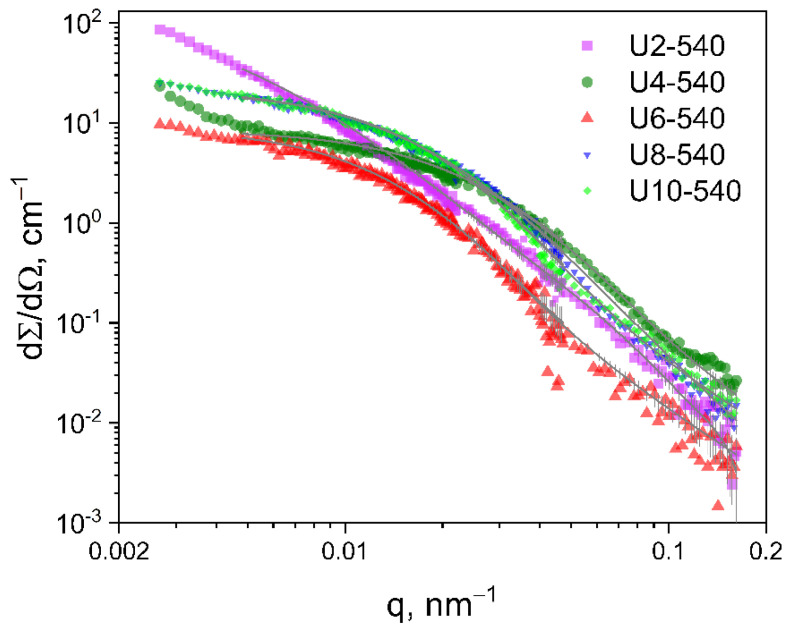
USANS data (symbols) and the fitted models of polydisperse spheres (lines).

**Figure 6:**
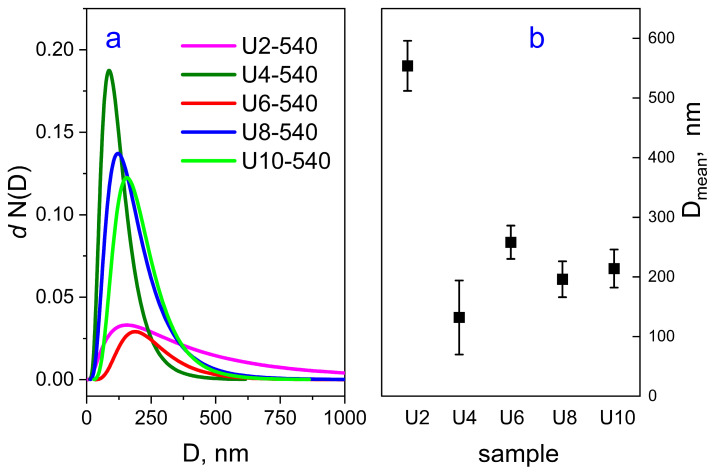
Fitted size distributions obtained from USANS measurements for submicron-sized particles (**a**) and their mean diameters obtained from the distribution (**b**).

The neutron scattering curves in the scattering vector (*q*) range 3 × 10^−3^–2 × 10^−2^ nm^−1^ show a pronounced Guinier region for all studied samples (see Figure 5). The simple model of spheres with a log-normal size distribution was used for analyzing the scattering entities with sizes of hundreds of nm. The resulting size distributions are shown in Figure 6a. The large *q* part of the experimental data did not align well with the fit by the unimodal polydisperse particle model; therefore, the addition of a population of small particles with sizes in the range of 1–100 nm was necessary to achieve a better fit. Such sizes are near or beyond the instrumental resolution of the USANS setup, which makes the calculated sizes rather uncertain. The obtained volume fractions of the small particles were about 1–2%; therefore, their contribution to the accuracy of the results for the larger, submicron-size particles was negligible.

The mean diameters of the scattering objects calculated from the fitted size distributions are shown in Figure 6b.

### 2.4. Nanoscale Structure

#### 2.4.1. Nitrogen Sorption

**Figure 7:**
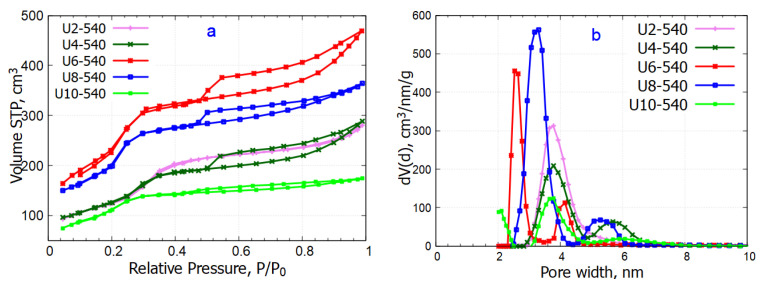
Nitrogen adsorption/desorption isotherms (**a**) and pore size distributions (**b**) for the calcined series of samples.

The N_2_ adsorption/desorption isotherms are shown in Figure 7a, and the calculated structural parameters are collected in Table 3. The materials present type IVa isotherms, specific for mesoporous materials, where capillary condensation is accompanied by hysteresis [27,28]. For sample U2-540, the hysteresis is very narrow, and the isotherm is more likely of IVb type. For the other four samples, the hysteresis loop is more pronounced and has an H2(b) shape, associated with pore blocking and pores of ink-bottle shapes [27]. The narrow hysteresis loop closing near 0.35 (sample U10) or 0.45 (U4, U6, U8) P/P_0_ suggests the presence of wide pores that are connected to the external surface [28].

The pore size distribution (Figure 7b) is unimodal and centered around 3.7 nm for the U2-540 sample. For the other four samples, the distribution appears to be bimodal, with the maximum of the smaller pores situated between 2.5 and 4.0 nm, and between 4.0 and 6.5 nm for the populations of the larger pores. In the sample U10, prepared using the gemini surfactant with the longest spacer, besides the mesopores, a small amount of micropores was also formed with a micropore area of 78 m^2^/g. The total pore volumes for all materials were in the 0.27–0.73 cm^3^/g range, showing the maximal value for the U6-540 sample.

#### 2.4.2. Transmission Electron Microscopy

**Figure 8:**
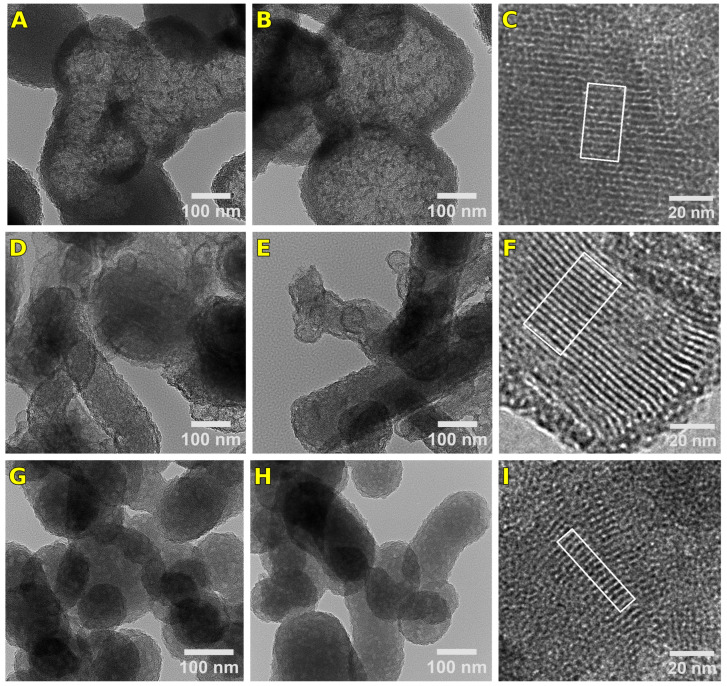
Characteristic TEM images of samples U2-540 (**A**–**C**), U6-540 (**D**–**F**), and U10-540 (**G**–**I**).

**Figure 9:**
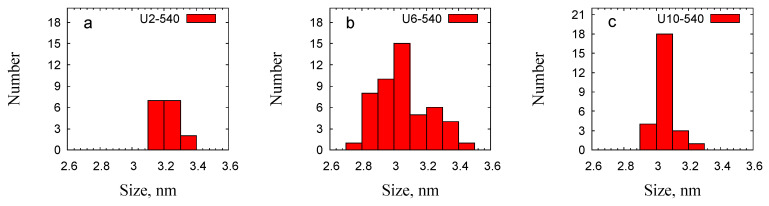
Interplanar distance distributions observed by TEM for the U2-540 (**a**), U6-540 (**b**), and U10-540 (**c**) samples.

TEM analysis was performed on three representative samples—U2-540, U6-540, and U10-540—prepared using urea-based gemini surfactants with the short, medium, and highest spacer lengths, respectively. All particles in the U2-540 sample were spherical, with certain particles displaying hollow structures characterized by a darker shell surrounding a lighter core (Figure 8A,B). In contrast, the U6-540 sample exhibited spherical and cylindrical particle shapes, with some cylindrical particles appearing curved (Figure 8D,E). The U10-540 sample predominantly consisted of spherical particles, with a few of cylindrical shape (Figure 8G,H). These morphologies agreed with the results of the SEM analysis performed over a much higher number of particles. The interplanar spacing of the hexagonal lattice was evaluated by selecting specific regions from the TEM images of all three samples. The interplanar distances measured using ImageJ software (version 1.54p) ranged across the three investigated samples between approximately 2.7 and 3.5 nm. The results were approximated by a Gaussian distribution, yielding a dominant periodicity of approximately 3.22 nm for U2-540, followed by about 3.05 nm for U10-540, and around 3.0 nm for U6-540. The broader distribution observed for U6-540 indicates a less uniform structure compared to the other samples (Figure 9). The interplanar spacings were the highest for the sample U2-540 (Figure 9).

#### 2.4.3. Small-Angle X-Ray Scattering

**Figure 10:**
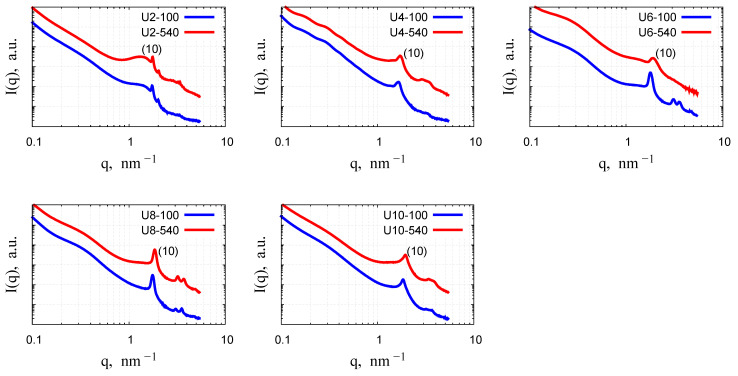
SAXS diffractograms of the mesoporous silica samples prepared with gemini surfactants of different spacer lengths.

The analysis of the structural ordering of all investigated samples was conducted using small-angle X-ray scattering. Figure 10 presents the small-angle diffraction patterns for the samples before and after calcination. All samples exhibit a characteristic feature: a steep decay in the small *q* range, attributed to scattering from particles of sizes larger than 100 nm, which is beyond the detection range of the instrument [29]. For the U4, U6, U8, and U10 samples, three peaks in the *q* range 1.5–4 nm^−1^ can be observed and assigned to the (10), (11), and (20) reflections of a two-dimensional lattice with the p6mm space group. These peaks indicate a highly ordered mesoporous structure characteristic of MCM-41 materials, where the pore arrangement is periodic although the silica walls are amorphous [11,13,15,29,30,31,32]. Out of those samples, the calcined U6-540 sample exhibits only a single, broadened peak, suggesting a significant loss of long-range order during the calcination process. This results from shrinkage and densification of the mesoporous framework caused by the high-temperature heat treatment, which shifts the (10) peak to higher *q* and, in some cases, leads to the suppression of higher-order peaks [15,30,31,32,33,34,35,36]. The hexagonal order is most pronounced for samples U6-100 and U8-100, as all three characteristic low-angle reflections are well resolved. The second and third peaks of the U4-100 and U10-100 samples are present but not strong or well-defined. However, after calcination, these two peaks become more noticeable, though they are not sharp enough to be clearly distinguished. This indicates that the hexagonal mesostructure is preserved, but the long-range periodicity is less pronounced compared to the U6 and U8 samples, as reflected by the lower intensity and broadening of higher-order reflections.

The U2 sample shows a markedly different structure: the combination of a broad peak around *q* = 1.2 nm^−1^ and sharper subsequent peaks may suggest that at least two distinct crystalline structures are present in the material.

**Figure 11:**
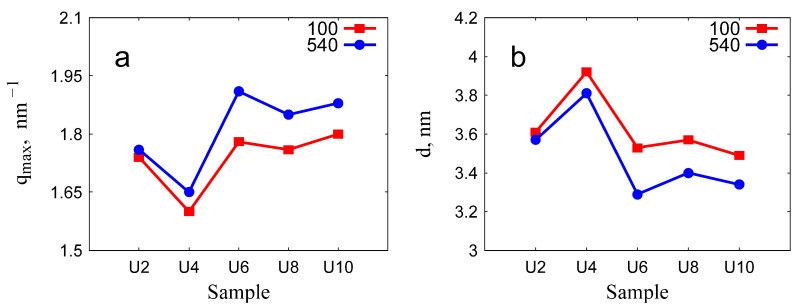
Position of the (10) reflection (**a**) and the lattice spacing (**b**) for the samples dried at 100 °C (red squares) and the samples calcined at 540 °C (blue circles).

The variation of peak positions and the corresponding lattice spacings with the surfactant spacer length is illustrated in Figure 11. The peak positions exhibited similar trends across materials prepared with surfactants of varying spacer lengths. Specifically, an increase in the spacer length resulted in a shift of the first diffraction peak towards higher *q* values.

### 2.5. Influence of the Surfactant Spacer Length

Gemini surfactants with urea groups in their alkyl chains form micellar aggregates in an aqueous environment [18]. In the basic synthesis conditions used in this study, the micellar pore templates led in all but one case to the formation of well-ordered MCM-41-type structures. The resulting particle morphologies were influenced by the specific surfactant used and displayed certain irregularity, with two predominant shapes observed: nearly spherical particles and elongated (cylindrical) forms. This behavior is markedly different from the OMS prepared using gemini surfactants with a diester spacer, for which the variation of the spacer length between two and eight methylene groups led to the growth of quasi-spherical particles of similar average size of about 600 nm [15]. For the silica prepared with the gemini surfactant with the shortest spacer, the diffraction shows the presence of two different phases, which can be related to the incomplete dissolution of the surfactant in the reaction medium, observed by small-angle neutron scattering [18].

The gemini surfactants with different spacer lengths affected the porosity of the particles: a trend of the decrease of the interplanar spacing with increasing the surfactant spacer length was revealed by the variation of the position of the first (10) diffraction peak. Calcination at 540 °C induced shrinkage of the unit cell dimension by about 3–7% (Table 4), resembling the structural change commonly observed in ordered mesoporous silica prepared with CTAB or other surfactants [15,30,31,32,33,34,35]. The U6-540 sample proved to have the least structural resistance, as seen by the loss of long-range order after calcination at 540 °C. This feature correlates well with the highest specific surface area and mesopore volume of this sample. The irregular variation of the lattice parameter and the particle morphology for OMS silica prepared with the surfactants of different spacer lengths can be related to the variability of the micellar aggregate shape, characteristic for the gemini molecules, either with very short poly(methylene) spacers (2–3 CH_2_ groups), or long flexible spacers [37,38]. For the gemini surfactants with urea groups in the alkyl tail, an intramolecular hydrogen bonding between the neighboring molecules can also influence the micelle shape and the micelle—silica interfaces during the condensation reaction.

### 2.6. Lattice Spacing and Domain Size

The TEM analysis enabled a quantitative assessment of structural irregularities in the hexagonal pore networks. Specifically, the analysis of the statistical distribution of lattice spacing values can provide a valuable insight into the morphology of the porous structure and the interpretation of the various parameters that describe the pore ordering. These real-space quantities are directly linked to the broadening of the diffraction peaks, and their contributions can be separated based on the numerical analysis of the TEM images.

The broadening of the SAXS peaks reflects the underlying structural disorder of the OMS, and can originate from three main factors: finite domain size, non-uniformity of the lattice spacing, and instrumental resolution. Assuming Gaussian profiles for each component, the total observed peak width $\sigma_{obs}$ can be described by the convolution relation [39]:

(1) $$\sigma_{obs}^{2}\;=\sigma_{size}^{2}\;+\sigma_{lattice}^{2}\;+\sigma_{ins}^{2}$$

Here, $\sigma_{size}$ represents broadening due to finite domain size, $\sigma_{lattice}$ due to lattice spacing variation, and $\sigma_{ins}$ due to instrumental resolution.

In our study, the peak broadening caused by the instrumental resolution was quantified by fitting the direct beam profile to a Gaussian function [40]. The standard deviation $\sigma_{ins}$, representing the instrumental resolution in reciprocal space, was found to be 0.0135 nm^−1^, determined by the divergence of the incoming beam, whereas the contributions of the detector pixel size and the beam monochromaticity are much smaller [41]. The instrumental resolution was incorporated into the analysis to ensure reliable separation of the broadening components.

The broadening caused by the lattice spacing variation was estimated directly from TEM. The presence of different spacing is due to the non-uniformity of the silica walls surrounding the micellar core. The thickness of the silica wall depends on the reaction speed during the condensation phase, which can be different at different locations due to the heterogeneous distribution of the reactants. These effects are rarely reported for OMS, presumably due to the difficulty of their determination. As the peak position *q* is inversely proportional to the interplanar spacing *d*, any variation in *d* introduces a spread in *q*. By propagating the variance through this relationship, the lattice-related broadening can be approximated as:

(2) $$\sigma_{lattice}\approx \frac{1}{d_{m}^{2}}\times \varepsilon_{d}$$

where $d_{m}$ (nm) is the average lattice spacing and $\varepsilon_{d}$ (nm) is the standard deviation obtained from the TEM *d*-spacing distribution (Figure 9).

Using Equation (1), the individual broadening contributions were separated. The total observed SAXS peak broadening $\sigma_{obs}$ was determined by Gaussian fitting of the diffraction peaks. The lattice spacing variation component $\sigma_{lattice}$ was calculated using Equation (2) based on the mean and standard deviation of interplanar spacings determined by TEM. The instrumental broadening $\sigma_{ins}$ was incorporated as described above. Finally, the residual broadening, attributed to finite domain size ($\sigma_{size}$), was isolated, allowing estimation of the structural coherence length—defined as the average distance over which the ordered pore structure remains intact.

Table 5 summarizes this analysis for two samples (U6-540 and U10-540). After correcting for both lattice spacing variation and instrumental resolution, the calculated domain sizes increased slightly for both samples. Specifically, for U6-540, the structural coherence length increased from 33.2 nm to 34.6 nm, and for U10-540, from 12.6 nm to 13.0 nm. These results indicate that in the studied materials, the SAXS peak broadening is primarily caused by the finite domain size of the ordered regions, with only minor contributions from lattice spacing variation. The effect of the instrumental line broadening is negligible when using the standard SAXS configurations.

## 3. Conclusions

This work demonstrated the applicability of cationic gemini surfactants having urea groups in their spacer chain to produce MCM-41-type mesoporous silica material in a sol–gel process, with TEOS as silica precursor, at basic conditions. The variation of the spacer length, which determines the structure and size of the micelles in water, led to the formation of a variety of spherical and elongated silica particles with at least one of their dimensions below 1 micrometer. A simultaneous formation of spherical and cylindrical silica particles at different proportions was found for the different numbers of methylene groups in the spacer. While the spacer length strongly affected the shapes and sizes of the nanoparticles, no regular pattern was observed between the length of the spacer and the characteristic morphology of the particles.

The variation in the spacer length affected both the submicron shapes and sizes of the nanoparticles and the morphology of the ordered pore structure on the nanometer scale. In the sample obtained with the shortest spacer gemini surfactant, two different crystalline structures were identified from the low-angle diffraction patterns, whereas the other four materials had the conventional 2D hexagonal pore structure, typical for OMS prepared with cationic surfactants. With the increase in the spacer length, a slight decrease in the lattice spacing was observed. The surfactant with six methylene units produced the OMS with the largest specific surface and pore volume; however, the degree of ordering of the pore structure was the lowest compared to the materials prepared with the gemini surfactants of shorter or longer spacer chains.

The application of high-resolution TEM and statistical analysis of the interplanar distances within the crystalline domains provided valuable insights into the morphology of the porous structure and the contribution of the effects responsible for the peak broadening. It has been shown that in the studied materials, the main source of the broadening of the diffraction peaks was the finite domain size, while the variation of the lattice spacing contributed only to about 25% of the total peak width.

## 4. Materials and Methods

### 4.1. Synthesis of Urea Gemini Surfactants

The synthesis is divided into two steps: preparation of the precursor and the synthesis of the urea-based gemini surfactant series.

The 1-isocyanatoundecane was prepared and freshly distilled before use. The urea-containing precursor—1-[2-(dimethylamino)ethyl]-3-undecylurea—was prepared as follows. Into the ice-cooled stirred solution of 1-isocyanotoundecane (10 g, 35 mmol) in 25 mL of dry acetonitrile, 2.1 g (24 mmol) of 2-(N, N-dimethyl)ethane-1,2-diamine was added dropwise over 15 min. Next, the solution was stirred for one hour, and the temperature was maintained between −5 and 0 °C. The crude residue was filtered and washed with anhydrous diethyl ether. The crude product was dried in a vacuum and was used in the next step without further purification.

The gemini surfactants were obtained via S_N_2 Menshutkin reaction as follows: 1 eq. of 1-[2-(dimethylamino)ethyl]-3-undecylurea was suspended in 20 mL of anhydrous acetonitrile, and 0.51 eq. of corresponding α,ω–dibromoalkane was added, and the mixture was refluxed for 24 h in anhydrous conditions. After cooling to room temperature, the solvent was evaporated in a vacuum, and the crude residue was crystallized from anhydrous acetone. The obtained powders were dried in a vacuum desiccator over P_4_O_10_. The NMR spectra of the surfactant series are provided in [18].

### 4.2. Synthesis of Mesoporous Silica Materials

The mesoporous silica materials were synthesized using the Stöber method modified for the preparation of silica with an ordered pore structure [19,20]. Tetraethoxysilane (TEOS, 99%, Merck, Darmstadt, Germany), ethanol, ammonia solution, and distilled water were used as received. Next, 0.15 g of the gemini surfactant was dissolved in a 20 mL/5 mL water/ethanol mixture. The mixture was incubated at 35 °C for 15 min in an oven and stirred for 30 min at room temperature. Then, 3.0 mL of aqueous ammonia (25%) was added dropwise under constant stirring. After 10 min, 1.5 mL of TEOS was added slowly under continuous stirring. The reactant amounts were 0.198–0.172 mmol surfactant, 6.8 mmol TEOS, 1.11 mol H_2_O, 86 mmol EtOH, and 38 mmol NH_3_. The reaction mixture was stirred for 3 h at ambient conditions to enable the formation of the silica network. In the alkaline conditions, the dissociated anionic silicate species are attracted to the positively charged headgroups of the surfactant molecules, and during condensation, facilitate the parallel alignment of the cylindrical micelles. Subsequently, polymerization proceeds and glassy SiO_2_ walls are formed between the elongated micelles that arrange in a 2D hexagonal lattice.

After 24 h of aging, the precipitated silica product was collected by vacuum filtration and washed repeatedly with distilled water. The washed solids were dried at room temperature and then in an oven at 100 °C. These samples were labelled as U*s*-100, “s” being the number of methylene groups in the spacer of the applied gemini surfactant. The surfactant was removed by calcination at 540 °C for 5 h (heating rate: 1 °C/min). The calcined samples were designated as U*s*-540.

### 4.3. Characterization

Fourier-transform infrared spectra were recorded on KBr pellets using a Cary 630 FTIR spectrophotometer (Agilent Technologies LDA, Santa Clara, CA, USA).

Nitrogen sorption analysis was performed using a Quantachrome Nova 1200e apparatus (Quantachrome Instruments, Boynton Beach, FL, USA). Prior to the analysis, the samples were degassed in a vacuum for 6 h. The specific surface area was determined by the Brunauer–Emmett–Teller (BET) method in the relative pressure range P/P_0_ 0.05–0.25. The micropore surface area and volume were determined using the V-t method in the relative pressure range 0.15–0.40. Pore size distribution was evaluated with the Density Functional Theory (DFT) equilibrium model in the range 0.05–1. The pore size was also determined by the Barrett–Joyner–Halenda (BJH) method from the adsorption and desorption branches. The total pore volumes were determined using the point closest to 1 for the relative pressure.

The TEM investigations were performed using a Cs corrected 200kV Themis (Thermo Fischer, Waltham, MA, USA) electron microscope. Powders were suspended in ethanol and placed on carbon-coated copper grids.

Scanning electron microscopy imaging was performed with a LEO 1540 XB instrument operated with low accelerating voltage (2 kV) and low beam current (40 pA). The powder was dispersed on a sticky carbon tape to provide better conductivity during the measurement.

Small-angle X-ray scattering measurements were performed with a SAXSPoint 2.0 instrument (Anton Paar Gmbh, Graz, Austria) equipped with an EIGER2 R 1M position-sensitive detector (Dectris, Dättwil-Baden, Switzerland). Powdered samples were confined between two sticky tapes on a plastic grid and mounted on an automatic sample changer. The detector distance was calibrated with known XRD peak positions of silver behenate.

Ultra-small-angle neutron scattering has been used to reveal the morphology of the nanosized and submicron-sized structural elements of the materials. The measurements were performed with the double-crystal diffractometer MAUD (version 2.92) [42] operating at the thermal channel of the LVR15 10 MW research reactor in Řež, Czech Republic. Samples were measured in quartz cuvettes of 2 mm flight path, and the data modeling was performed using the SASProFit software (version 5.0.5.1.).

## Figures and Tables

**Table 1:** The assignment of the IR vibration bands in the calcined samples.

Band Assignments	υ [cm^−1^]
Free silanol groups and molecular water [21]	3455
Molecular water and the SiO_2_ network [22]	1636
Asymmetric Si–O–Si stretching [23]	1088
Si–OH stretching [24,25,26]	961
Symmetric Si–O–Si stretching [23,24]	806
Si–O–Si bending [21,23]	463

**Table 2:** Characteristics of particle morphologies and distributions of the particle sizes and shapes according to the quantitative analysis of the SEM images.

Sample	Morphology	Diameter Range [nm]
U2-100	100% Spherical	Spherical: 100–800
U4-100	50% Spherical, 50% Cylindrical	Spherical: 25–325, Cylindrical: 45–105
U6-100	50% Spherical, 50% Cylindrical	Spherical: 50–550, Cylindrical: 75–275
U8-100	35% Spherical, 65% Cylindrical	Spherical: 65–145, Cylindrical: 65–195
U10-100	85% Spherical, 15% Cylindrical	Spherical: 75–325, Cylindrical: 110–230

**Table 3:** Surface area, pore volume, and pore diameter for the calcined mesoporous silica samples determined by low-temperature nitrogen sorption.

Sample	Specific Surface Area [m^2^/g]	Specific Micropore Area [m^2^/g]	Pore Diameter [nm]			Total Pore Volume [cm^3^/g]	Micropore Volume [cm^3^/g]
			BJH(ads)	BJH(des)	DFT		
U2-540	481	-	3.1	3.3	3.8	0.43	-
U4-540	464	-	3.5	4.3	3.8	0.45	-
U6-540	910	-	3.1	4.0	2.7	0.73	-
U8-540	882	-	3.4	4.0	3.3	0.56	-
U10-540	443	78	3.1	3.1	3.8	0.27	0.02

**Table 4:** Characteristic parameters of the pore structure of the mesoporous silica samples determined by SAXS and TEM.

Sample	(10) Peak Position [nm^−1^]	HWHM by SAXS [nm^−1^]	d [nm]	Domain Size [nm]	Reduction in Domain Size	d and HWHM by TEM [nm]
U2-100	1.74	0.06	3.61	103.8		
U2-540	1.76	0.05	3.57	118.6	0.87	3.22, 0.09
U4-100	1.60	0.45	3.92	13.18		
U4-540	1.65	0.52	3.81	11.38	0.86	
U6-100	1.78	0.16	3.53	36.11		
U6-540	1.90	0.18	3.31	33.22	0.92	3.02, 0.16
U8-100	1.76	0.12	3.57	48.85		
U8-540	1.85	0.16	3.40	37.75	0.77	
U10-100	1.80	0.36	3.49	16.28		
U10-540	1.88	0.47	3.34	12.58	0.77	3.05, 0.05

**Table 5:** The components of the peak broadening for the (10) reflection in the SAXS diffractograms, and their influence on the determination of the domain size.

Sample Name	*σ_obs_* [1/nm]	*σ_lattice_* [1/nm]	*σ_ins_* [1/nm]	*σ_size_* [1/nm]	Domain Size After the Correction [nm]	Domain Size Before the Correction [nm]
U6-540	0.075	0.018	0.013	0.072	34.6	33.2
U10-540	0.2	0.005	0.013	0.199	13.0	12.6

## Data Availability

Experimental data associated with this article are available at https://hdl.handle.net/21.15109/ARP/N3JA2D (accessed on 3 October 2025) and https://doi.org/10.5158/ARP/N3JA2D (accessed on 3 October 2025).
